# Micropatterned Poly(D,L-Lactide-Co-Caprolactone) Conduits With KHI-Peptide and NGF Promote Peripheral Nerve Repair After Severe Traction Injury

**DOI:** 10.3389/fbioe.2021.744230

**Published:** 2021-12-09

**Authors:** Xing Yu, Deteng Zhang, Chang Liu, Zhaodi Liu, Yujun Li, Qunzi Zhao, Changyou Gao, Yong Wang

**Affiliations:** ^1^ Department of Thyroid Surgery, The Second Affiliated Hospital, Zhejiang University School of Medicine, Hangzhou, China; ^2^ MOE Key Laboratory of Macromolecular Synthesis and Functionalization, Department of Polymer Science and Engineering, Zhejiang University, Hangzhou, China; ^3^ College of Medicine, Zhejiang University, Hangzhou, China

**Keywords:** severe traction injury, biodegradable polyester, nerve guidance conduit, KHIFSDDSSE peptide, nerve growth factor

## Abstract

Severe traction injuries after stretch to peripheral nerves are common and challenging to repair. The nerve guidance conduits (NGCs) are promising in the regeneration and functional recovery after nerve injuries. To enhance the repair of severe nerve traction injuries, in this study KHIFSDDSSE (KHI) peptides were grafted on a porous and micropatterned poly(D,L-lactide-co-caprolactone) (PLCL) film (MPLCL), which was further loaded with a nerve growth factor (NGF). The adhesion number of Schwann cells (SCs), ratio of length/width (L/W), and percentage of elongated SCs were significantly higher in the MPLCL-peptide group and MPLCL-peptide-NGF group compared with those in the PLCL group *in vitro*. The electromyography (EMG) and morphological changes of the nerve after severe traction injury were improved significantly in the MPLCL-peptide group and MPLCL-peptide-NGF group compared with those in the PLCL group *in vivo*. Hence, the NGCs featured with both bioactive factors (KHI peptides and NGF) and physical topography (parallelly linear micropatterns) have synergistic effect on nerve reinnervation after severe traction injuries.

## Introduction

The peripheral nerve traction injury is one of the most common iatrogenic injuries in clinic (Schwartzman and Grothusen, 2008). The traction-related injury of the peripheral nerve, including brachial plexus, recurrent laryngeal nerve, and sciatic nerve, results in neuropathic pain, dysfunction of sensory, and motor systems (Liu H. et al., 2020). The tractive damage on the nerve takes place mainly at the outer epineurium and perineurium, whereas the structure inside the endoneurium remains relatively intact (Chiang et al., 2008). These morphologic findings suggest that the nerve palsies from traction injuries are temporary, and the electromyography (EMG) would gradually gain partial recovery (Brauckhoff et al., 2018). However, if the traction is prolonged or repeated, the resulted nerve root avulsion or widespread longitudinal damage would restrict the EMG recovery due to the limited possibilities for nerve reconstructions (Malessy et al., 2004). The current standard of nerve repair for severe traction injury is autografting (Zheng et al., 2018). This procedure involves the harvest of a section of donor nerve, and the transplantation into the defect (Ray and Mackinnon, 2010). However, the autografting will cause an additional surgery to excise the peripheral nerve, which increases the chance of surgical complications. Additionally, the donor nerve may be insufficient, and the autografts are also restricted to the diameter mismatch between the donor and acceptor nerves (Cheah et al., 2017). Hence, there is a strong demand to find a new way for nerve repair after severe traction injury.

The nerve guidance conduits (NGCs) have shown great promise in the regeneration and functional recovery of peripheral nerves (Zhang et al., 2020a). However, NGCs are rarely used for the nerve traction injury repair, and the underlying impact needs to be elucidated. The morphologic characteristics of nerve traction injury are different from the transection or thermal injury, which are described as neural fiber nonlinearity, myelin degeneration, and internal structural collapse (Gluck et al., 2018). Although at a later stage the nerves experience partial structural and myelin regeneration, the continuity, linearity, and density of myelin cannot fully be recovered (Lopez-Silva et al., 2021). To create an appropriate microenvironment for nerve regeneration, a graphene oxide/poly(D,L-lactide-co-caprolactone) (PLCL) nerve conduit has been developed (Zhang et al., 2020b). Besides the surface chemical property, the surface morphology of NGCs is sculptured by longitudinally oriented structures, which has been found helpful in the orientation and motility of nervous system cells (Zhang et al., 2018a). Moreover, neural recovery can be also improved by using bioactive molecules such as cell growth factors and peptides (Xia et al., 2017; Sarker et al., 2018). For instance, KHIFSDDSSE (KHI) peptides, derived from neural cell adhesion molecules (NCAM), are immobilized onto a substrate, showing efficiency in selective guidance of migration of Schwann cells (SCs) (Ren et al., 2015).

**FIGURE 1:**
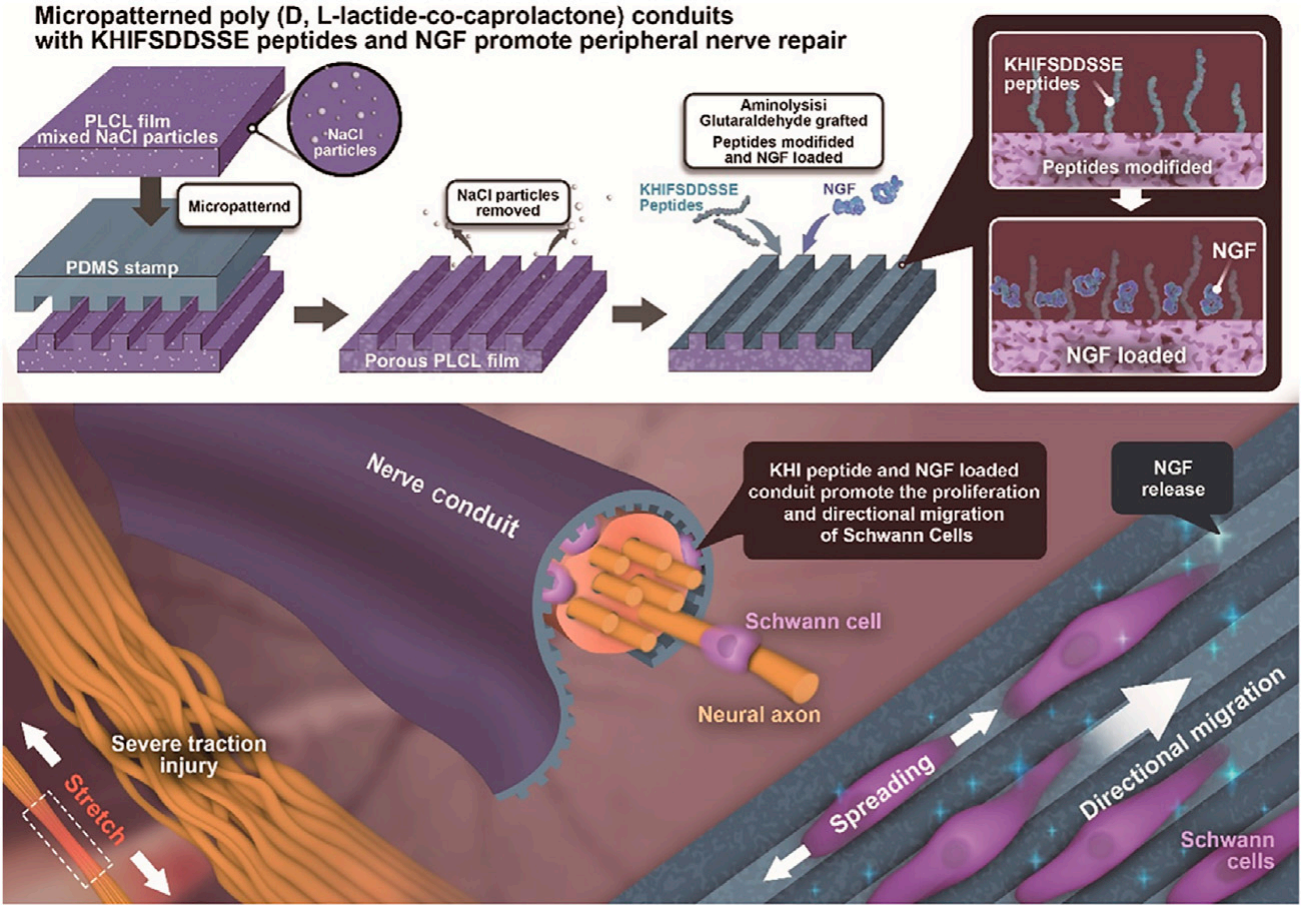
Illustration of PLCL NGC fabrication, KHI peptide modification, and NGF loading and its application in nerve reinnervation after traction injury. The PLCL film mixed with NaCl porogen particles is endowed with a micropattern structure by pressing a micropatterned PDMS stamp under heating. After rinsing out the NaCl particles, the porous micropatterned PLCL film is aminolyzed to introduce free amine groups and is then successively grafted with KHI peptides and NGF *via* glutaraldehyde coupling and/or physical adsorption. The Schwann cells are guided along the micro-stripes *in vitro*. The recovery of the sciatic nerve is enhanced by the functionalized PLCL NGCs *in vivo*.

In this study, a porous PLCL film with longitudinally oriented structure and KHI peptides/nerve growth factor (NGF) loading is designed to explore its applicability for the nerve repair after traction injury. The PLCL film mixed with NaCl particles is micropatterned by using a polydimethylsiloxane (PDMS) stamp under heating. After removal of the NaCl particles *via* water rinsing, the obtained porous micropatterned PLCL film is aminolyzed and grafted with the KHI peptides *via* glutaraldehyde coupling. The NGF is grafted/loaded on the film by reaction with the unreacted aldehyde groups and physical adsorption. The SCs are cultured on the modified PLCL film to investigate their adhesion, ratio of length/width (L/W), and elongation behavior (Figure 1). Moreover, the NGCs manufactured from the modified PLCL film are used in the sciatic nerve repair *in vivo*, whose overall performance is evaluated by EMG analysis, morphological observation, and neuromuscular junction (NMJ) during the process of reinnervation after traction injury.

## Materials and Methods

### Materials

PLCL with two different molar ratios of L-lactide and ε-caprolactone copolymers (50: 50, Mw = 50 kDa and 75: 25, Mw = 25 kDa) were purchased from Daigang Biotech (Jinan, China). Trichloromethane, methanol, acetone, NaCl, tertiary butanol, isopropyl alcohol, 1,6-hexanediamine, formaldehyde, ethanol, glutaraldehyde, and paraformaldehyde were supplied by Sinopharm Chemical Reagent (Shanghai, China). Ninhydrin hydrate (98%) and 5,5′-dithiobis-(2-nitrobenzoic acid) (DTNB) were purchased from Macklin Biochemical Co., Ltd. (Shanghai, China). KHIFSDDSSEK-Pra (KHI, Pra: L-C-propargylglycine) peptides were synthesized by Sangon Biotech (Shanghai, China). The water used in all the experiments was purified by a Milli-Q cycle purification system (Millipore, Bedford, MA, USA).

### Fabrication of Porous Micropatterned PLCL Films With Peptide Modification and NGF Loading

As described in our previous study (Zhang et al., 2018b), PLCL 50: 50, 0.07 g, and PLCL 75: 25, 0.03 g, were both dissolved in 2.8 ml trichloromethane under stirring for 12 h at room temperature. Then, the NaCl porogen particles, which were ground in a mortar and sieved through a 200-mesh sieve, were added into the PLCL solution, followed by a 1-h stirring. The mixed solution was cast onto a polytetrafluoroethylene (PTFE) mould to obtain the original PLCL/NaCl film after volatilization of the solvent. The micropatterns were created on the surface of PLCL film according to the following procedures. 1) A PDMS template was cross-linked with microstructures of grooves and ridges (4–5 μm in depth, and 5/5 μm in width) by copying from a micropatterned silicon wafer. 2) The PDMS stamp was pressed on the PLCL/NaCl films at 170°C to obtain the micropatterned surface of 5/5 μm in groove width/ridge width. 3) The micropatterned film was immersed in water for 3 days to remove NaCl particles to obtain the macropores. 4) The porous and micropatterned film was aminolyzed in 10% (v/v) 1,6-hexanediamine/isopropanol solution at 37°C for 3 min and then reacted with glutaraldehyde, followed by grafting of KHI peptides. Glutaraldehyde was washed in PBS (pH 7.2) for 3 times, each for 15 min (Abay et al., 2019). 5) The obtained film was immersed in 10 μg/ml NGF water solution to chemically graft/physically adsorb NGF *via* the remained aldehyde groups and porous structure under negative pressure, respectively. The cumulative release amount of NGF was detected by ELISA at 1, 3, and 7 days after NGF loading.

The surface morphology of the films was observed and verified by a scanning electron microscope (SEM, Hitachi S-4800, Tokyo, Japan). The amount of the -NH_2_ group on the aminolyzed PLCL films was detected by a ninhydrin assay method (Zhu et al., 2013). In order to measure the density of the peptides on the PLCL film, a calibration curve was firstly obtained by detecting the absorbance of various concentrations of peptides reacted with the DTNB, according to which the peptide density was calculated.

### SC Culture *in vitro*

SCs (from Sprague Dawley rat) were supplied by the Cell Bank of Typical Culture Collection of Chinese Academy of Sciences (Shanghai, China) and cultured in high-glucose Dulbecco’s modified Eagle’s medium (DMEM, Gibco, Grand Island, NY, USA) supplemented with 10% (v/v) fetal bovine serum (FBS, Sijiqing Inc., Hangzhou, China), 100 U/ml penicillin, and 100 μg/ml streptomycin at 37°C in a 5% CO_2_-humidified cell incubator. The PLCL films were soaked in 75% ethanol for 15 min and washed 3 times with phosphate-buffered saline (PBS).

### SC Morphology

SCs were seeded onto the PLCL films at a density of 2 × 10^4^ cells/cm^2^ for 12 h and then freeze-dried after being fixed and dehydrated in gradient absolute ethanol/tertiary butanol for morphology detection using SEM. The L/W ratio and number of elongate SCs were measured by the Image-Pro Plus software.

### Animal Experiments

Thirty-two male Sprague-Dawley rats weighing 200–220 g were randomly divided into 4 groups (8 animals in each group). The corresponding films were reeled onto a steel bar (diameter 1.5 mm) in advance and then surrounded onto the surface of injured nerves. The NGCs were obtained *in situ* by sewing with 8–0 nylon monofilament suture stitches, where the injured nerve was ensured to locate at the middle of NGCs. The length of the NGCs was 12 mm, and the inner diameter and wall thickness were 1.5 and 0.2 mm, respectively (Zhang et al., 2020a). The groups were repaired with PLCL film (PLCL group), parallelly linear micropatterned PLCL film (MPLCL group), micropatterned PLCL film with KHI peptides modification (MPLCL-peptide group), and micropatterned PLCL film grafted with KHI peptides and loaded with NGF (MPLCL-peptide-NGF group), respectively. All the animal experiments were carried out in accordance to the institutional animal care guidelines. The procedures were reviewed and approved by the Institutional Review Board (Ethics Committee, the Second Affiliated Hospital of Zhejiang University, School of Medicine).

The rats were anesthetized by intraperitoneal injection of 3% pentobarbital saline (0.1 ml/100 g). The left sciatic nerve was dissected and exposed by dorsal gluteal muscle splitting. The nerve was continuously stretched by a tension meter (Ai Debao, Wenzhou, China) at 3.5 N for 30 s. The electrophysiological (EMG) evaluation and gross and microscopic observation (optical and electron microscope) were performed to confirm the establishment of the traction injury model.

### EMG Analysis

As the protocol described before (Yu et al., 2021), the simulating cathode composed of a stainless-steel monopolar needle was placed at the sciatic nerve trunk, with a parallel distance of 10 mm between the two cathodes. The motor response was recorded distally with a unipolar steel needle electrode inserted into the gastrocnemius muscle. The compound motor action potential (CMAP) and nerve conduction velocity (NCV) were recorded using a digital neurophysiological system (Neuro-MEP-Micro, Neurosoft Ltd., 5, Voronin str., Ivanovo, 153032, Russia).

### Transmission Electron Microscopy

Transmission electron microscopy (TEM) was used to record the changes in the nerve’s structure. The resected part of the nerve was fixed in 2.5% glutaraldehyde overnight in a refrigerator and washed in PBS (pH 7.2) for 3 times, each for 15 min. It was then placed in 1% osmium tetroxide for 1 h. After being stained in 4% uranyl acetate for 30 min, the sample was dehydrated by a series of concentrations of ethanol (70%, 80%, 90%, 95% ethanol, 10 min each) and embedded in a resin mixture. After sectioned, the sample was observed under a Tecnai 10 transmission electron microscope (Philips, Amsterdam, Netherlands) (Zhang et al., 2020b).

### Statistical Analysis

The results are presented as number (%) and average ±SD appropriate. Data were analyzed by one-way ANOVA, Welch ANOVA, Student *t*-test, the χ2 test, Fisher’s exact test, and non-parametric Wilcoxon–Mann–Whitney test appropriately using SPSS 20.0 software (SPSS Inc., Chicago, IL, USA). A *p* value less than 0.05 was considered to be statistically significant.

## Results

### Fabrication and Characterization of Micropatterns on the Surface of PLCL Films

**FIGURE 2:**
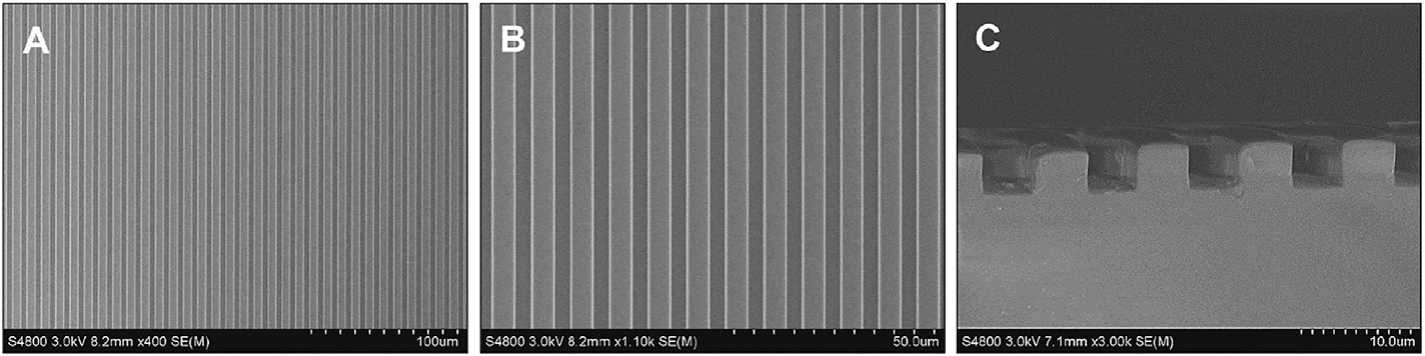
SEM images showing the micropatterns of ridges/grooves of 5/5 μm **(A,B)** and depth of 4–5 μm **(C)**.

The guidance of directional migration of SCs is helpful to the functional recovery from severe damage in the peripheral nerve system, yet the application in practice still remains challenging despite of some pioneering studies (Zhang et al., 2018a). Inspired by the highly aligned microstructure of native nerve fibers, organized topographical guidance and bioactive molecules have been introduced to the inner wall of various types of NGCs to enhance their nerve regeneration (Huang et al., 2020). Linear micropatterns can induce the elongation of SC and nerite orientation of dorsal root ganglion (Zhang et al., 2020a). Therefore, in this study the linear micropatterns were created firstly on the PLCL film by using a stamp made from PDMS, which has very low surface energy for anti-adhesion. Figure 2 shows that the micropatterns were regular and anisotropic, and the parameters of grooves and ridges were replica of the stamp with features of 4–5 μm in depth and 5/5 μm in width.

**FIGURE 3:**
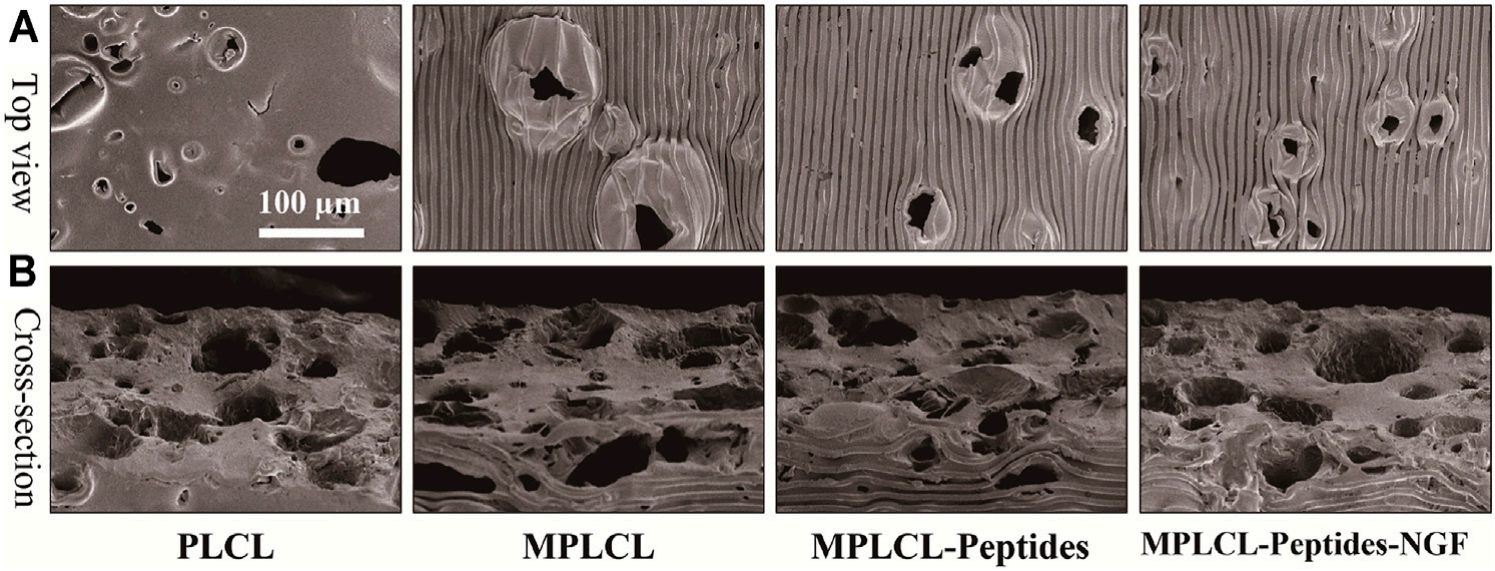
Top-view **(A)** and cross-sectional **(B)** SEM images of different PLCL films, including the groups of PLCL, micropatterned PLCL (MPLCL), micropatterned PLCL grafted with KHI peptides (MPLCL-peptides), and micropatterned PLCL grafted with KHI peptides and loaded with NGF (MPLCL-peptides-NGF).

Next, the same technology was applied to the PLCL film having NaCl particles with a diameter of 75.6 ± 19.1 μm (Supplementary Figure S1) to obtain a porous micropatterned PLCL film after porogen leaching. The topographical morphology of the PLCL film was observed by SEM. The film shows a plenty of pores on both the surface and interior (Figure 3A) with a diameter of ∼63.6 ± 29.9 μm (Figure 3B), regardless of the further grafting/loading of KHI peptides and NGF. Although the micropatterns were destroyed at the porous regions on the surface, the overall linear pattern structure was well maintained, which is important in guiding the spreading and elongation of cells contacted. After a series of surface treatment, the linear stripes on the film in each group were still maintained. Quantitative analysis found that the amount of -NH_2_ was 7.3 μg/cm^2^, and the density of KHI was 4.3 μg/cm^2^. NGF can accelerate the process of nerve repair and regeneration, and its release plays a vital role for axon stretching. As shown in Supplementary Figure S2, the release of NGF from the film increased along with time prolongation at least for 1 week, with a total released amount of 35 ng.

### Adhesion and Elongation of SCs *in Vitro*

**FIGURE 4:**
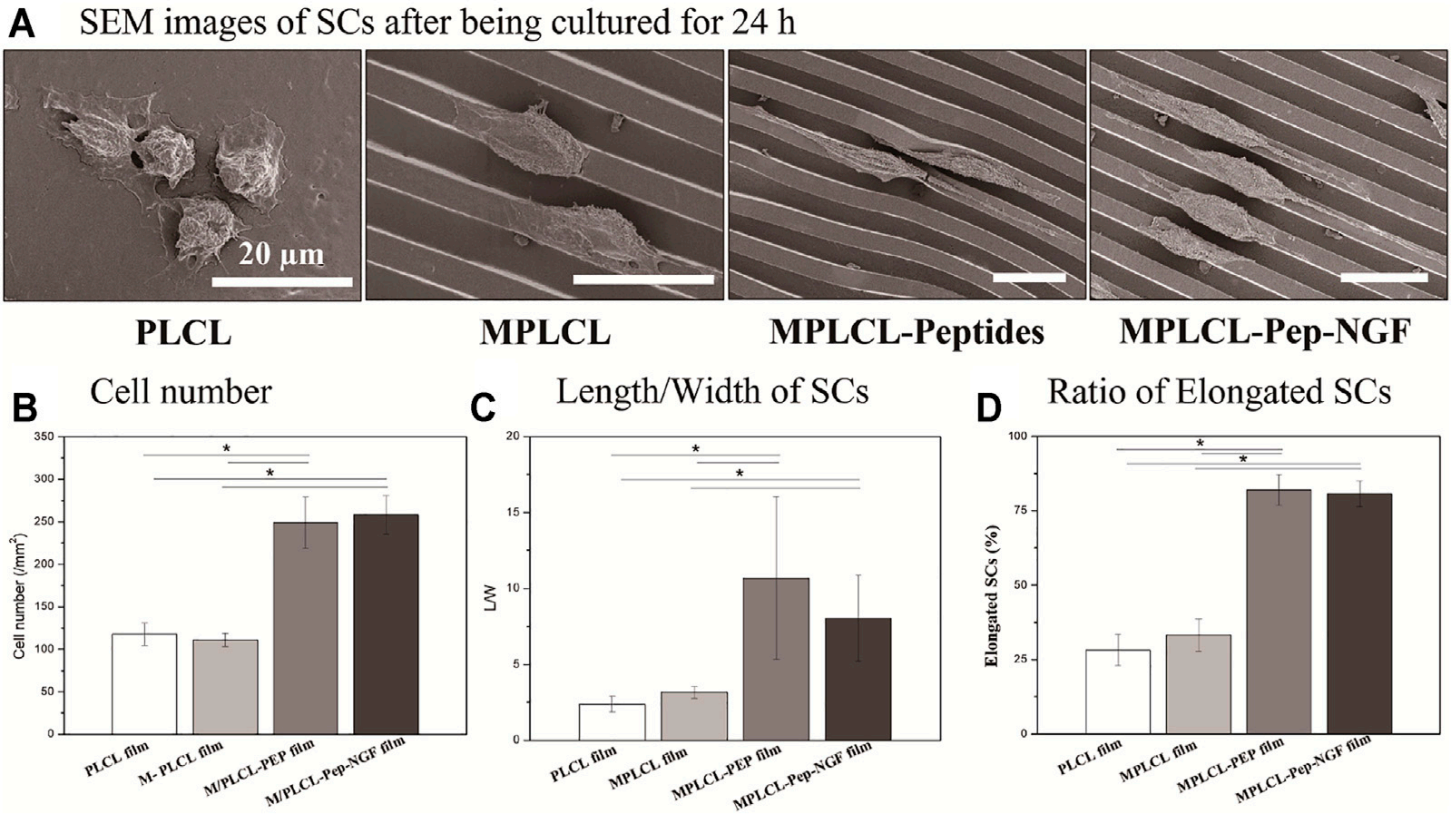
The growth status of SCs on the different PLCL films, including the groups of PLCL, MPLCL, MPLCL-peptides, and MPLCL-peptides-NGF. **(A)** SEM images of SCs after being cultured for 24 h. **(B)** The statistical data of cell number. **(C)** The length/width (L/W) of SCs. **(D)** The ratio of elongated SCs. Asterisk (*) indicates statistically significant difference at the *p* < 0.05 level, *n* = 3.

SCs, the main glial cells in the peripheral nervous system, take a critical role during nerve reconstruction. After the nerve injury, SCs proliferate and migrate to form the Bunger bands and to guide the newborn axon to bridge the two injured stumps. The affinity and elongation of SCs on the surface of NGCs directly decide the outcomes of the functional recovery. As shown in Figure 4A, SCs spread slightly with a round shape and random direction in the PLCL group. However, the cells tended to elongate along the microgrooves in the MPLCL group. By contrast, the cells showed a lathy shape in the MPLCL-peptide group and MPLCL-peptide-NGF group.

The adhesion numbers of SCs (∼250) in the MPLCL-peptide group and MPLCL-peptide-NGF group were significantly larger than that in the PLCL group (*p* < 0.05) (Figure 4B). As the irregular misconnection leads to functional disorder during the repair process, the regularly elongated and orientated cells can better guide the direction of newborn axons. The elongation factor, a parameter displaying the cell morphology index, is defined as the L/W ratio of SCs. The ratio of L/W was significantly increased in the MPLCL-peptide group and MPLCL-peptide-NGF group compared with that in the PLCL group (*p* < 0.05) (Figure 4C). The percentages of the elongated SCs in the MPLCL-peptide group and MPLCL-peptide-NGF group were significantly larger than that in the PLCL group as well (*p* < 0.05) (Figure 4D).

### Animal Evaluation

To evaluate the effects of KHI peptide grafting, NGF loading, and micropatterns *in vivo*, the PLCL film (PLCL group), micropatterned PLCL film (MPLCL group), micropatterned PLCL grafted with KHI peptides (MPLCL-peptide group), and micropatterned PLCL film grafted with KHI peptides and loaded with NGF (MPLCL-peptide-NGF group) were implanted to wrap the tractive injury points, respectively. All the animals were raised in the same environment for 4 weeks. The nerve conduction ability was detected by EMG analysis, and the morphology changes of nerves and gastrocnemius muscles (representing the recovery of reinnervation) were evaluated by optical and electron microscopy.

#### The Animal Model of Nerve Severe Traction Injury

**FIGURE 5:**
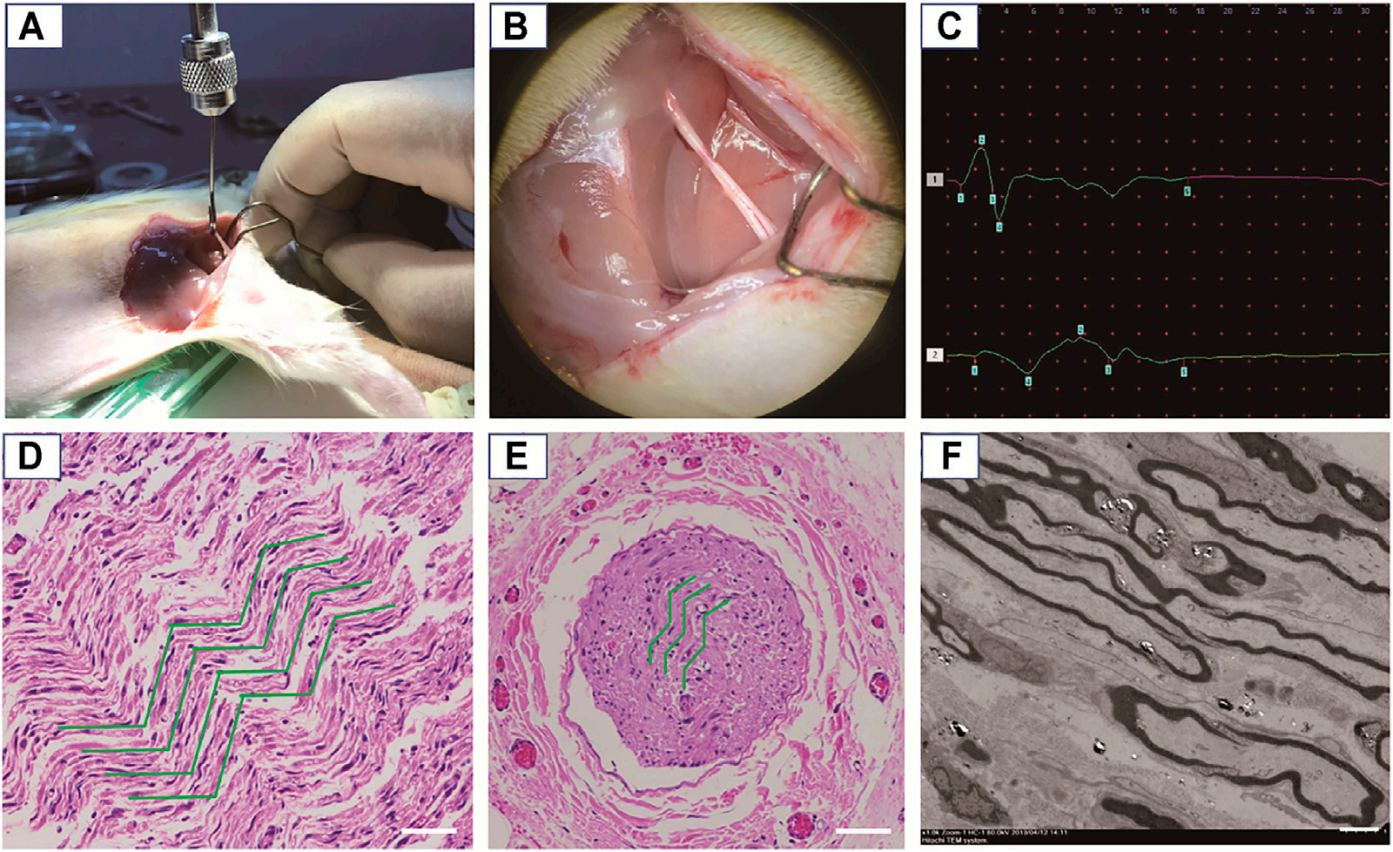
Establishment and confirmation of an animal model for nerve severe traction injury. **(A)** The nerve was continuously stretched at 3.5 N for 30 s. **(B)** Gross observation of the damaged nerve, exhibiting edema and congestion. **(C)** The representative CMAP results after traction injury (amplitude decrease > 50%, or latency prolongation > 10%). **(D)** The damaged nerve point was observed by an optical microscope after H&E staining, where the green curves show circuitous axons in the longitudinal section. Scale bars: 50 μm. **(E)** The damaged nerve point was observed by an optical microscope after H&E staining, where the green curves show the circuitous axons in the cross section. Scale bars: 200 μm. **(F)** TEM observation of the circular axons and myelin sheathes in cross section being stretched as elliptical appearance. Scale bars: 5 μm.

To establish the animal model of the nerve severe traction injury, the sciatic nerve in one side was continuously stretched at 3.5 N for 30 s. The successful establishment of animal models was confirmed by nerve congestive gross observation, decreased NCV, and the representative CMAP results (amplitude decrease > 50%, or latency prolongation >10%). Hematein and eosin (H&E) staining showed that the injured nerve exhibited nonlinear internal fibers and increased the fiber spacing apart from a distinct region of wavy. The structural damages were also observed as fiber shredding and myelin sheathe degradation (Figure 5).

#### EMG Analyses

**FIGURE 6:**
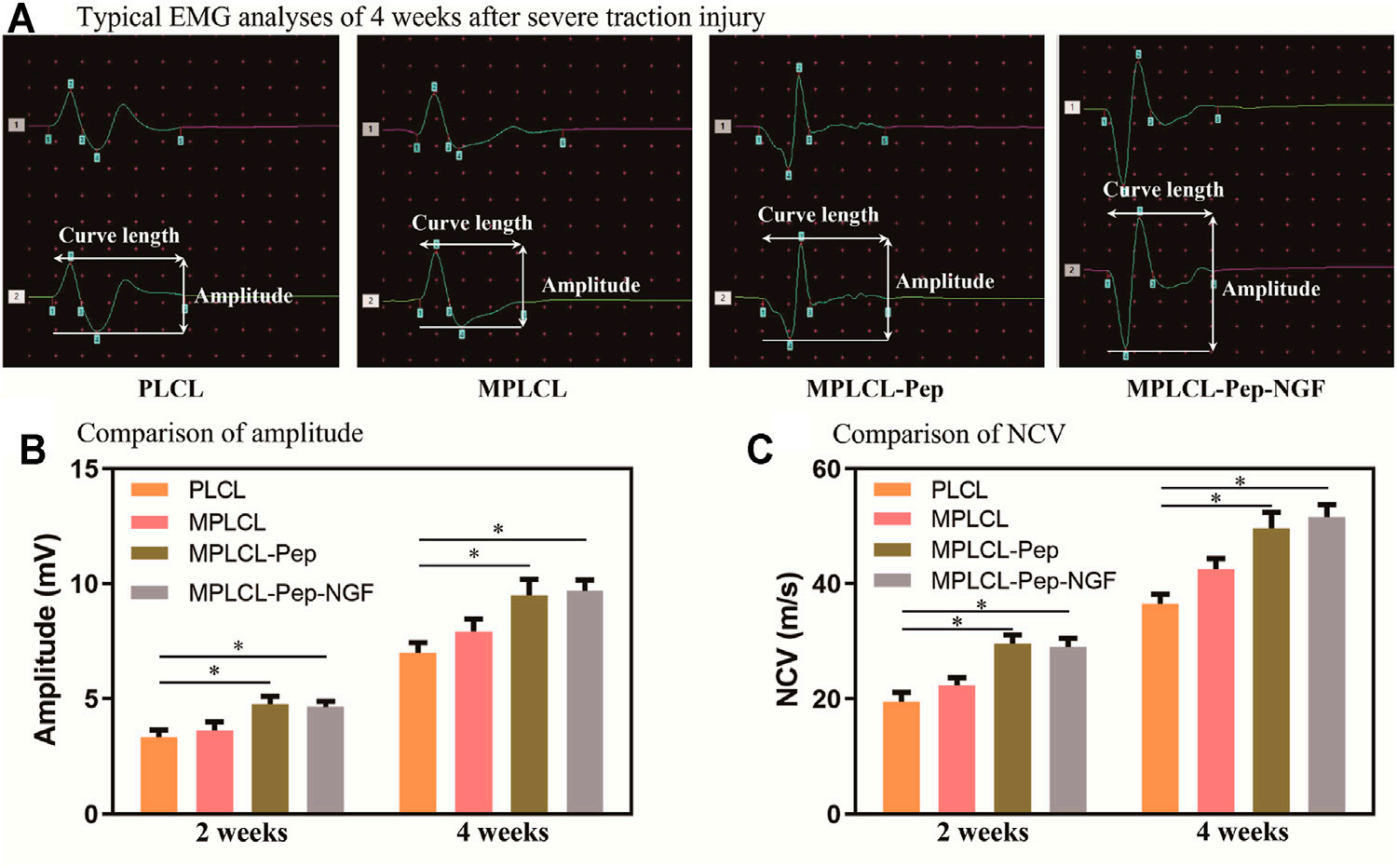
Neural conductive abilities were shown in the groups of PLCL, MPLCL, MPLCL-peptides, and MPLCL-peptide-NGF groups, respectively. **(A)** Typical EMG analyses of 4 weeks after severe traction injury. **(B)** The comparison of amplitude. **(C)** The comparison of NCV. Asterisk (*) indicates statistically significant difference at the *p* < 0.05 level, *n* = 8.

To evaluate the recovery of nerve conduction ability, EMG analyses (including CMAP and NCV) were performed in each treatment group at 2 and 4 weeks postoperatively. The representative waveforms for typical biphasic CMAP responses at 4 weeks postoperative for each treatment group are shown in Figure 6A. The CMAP amplitude was significantly increased in the MPLCL-peptide group and MPLCL-peptide-NGF group compared with that in the PLCL group at 2 and 4 weeks postoperative, respectively (*p* < 0.05) (Figure 6B). The NCV was also increased significantly in the MPLCL-peptide group and MPLCL-peptide-NGF group compared with that in the PLCL group at 2 and 4 weeks postoperative, respectively (*p* < 0.05) (Figure 6C).

#### Nerve Morphological Changes

**FIGURE 7:**
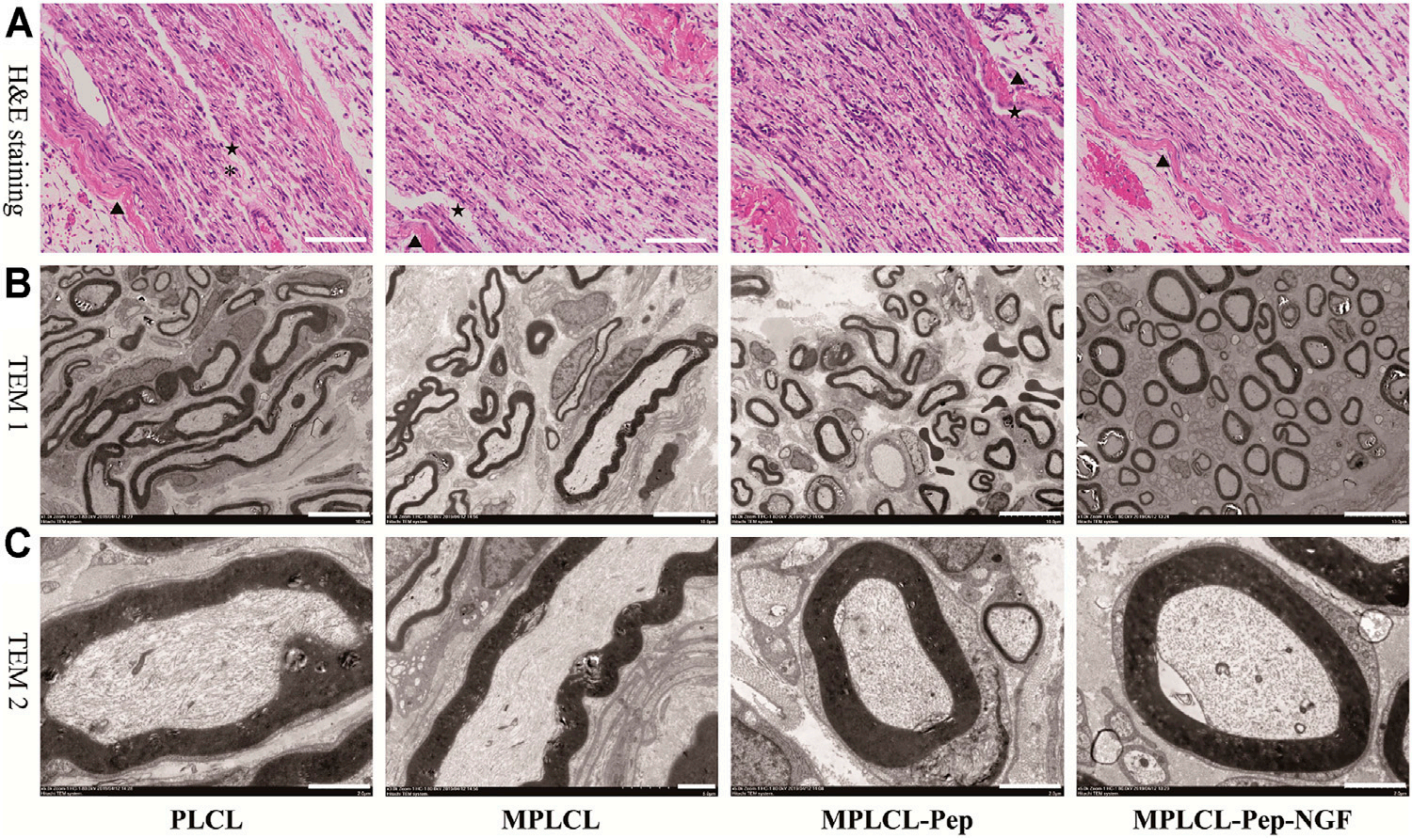
The typical nerve morphological changes in each treatment group of PLCL, MPLCL, MPLCL-peptides, and MPLCL-peptides-NGF. **(A)** H&E staining of the neural in the longitudinal section, where ▲, *, and ★ refer to the circuitous structure of epineurium, the circuitous structure of axons, and the fracture of axons. Scale bars: 100 μm. **(B)** Cross-sectional TEM images showing the circular or elliptical appearance of axons. Scale bars: 10 μm. **(C)** Cross-sectional TEM images showing axons and myelin sheathes. Scale bars: 2 μm.

The nerve morphological changes were observed by optical and electron microscopy at 4 weeks after NGC implantation (Figure 7). In the view of optical microscopy, the nonlinear structures at the injured point of axon fibers were alleviated in each treatment group. In the PLCL group, irregular structures (long, narrow, and wavy) of myelin sheath with lessoned anchoring particles were observed, accompanied with some fractures on the myelin sheath. By contrast, in the MPLCL-peptide group and MPLCL-peptide-NGF group, the remained fiber spacing was reduced, and the ratio of fiber fragment was significantly decreased. TEM images show circular myelinated axons and a thick myelin sheath, where the anchoring particles were observed for maintaining the integrity of the myelin sheath in the MPLCL-peptide group and MPLCL-peptide-NGF group.

#### Morphological Changes of Gastrocnemius Muscle

**FIGURE 8:**
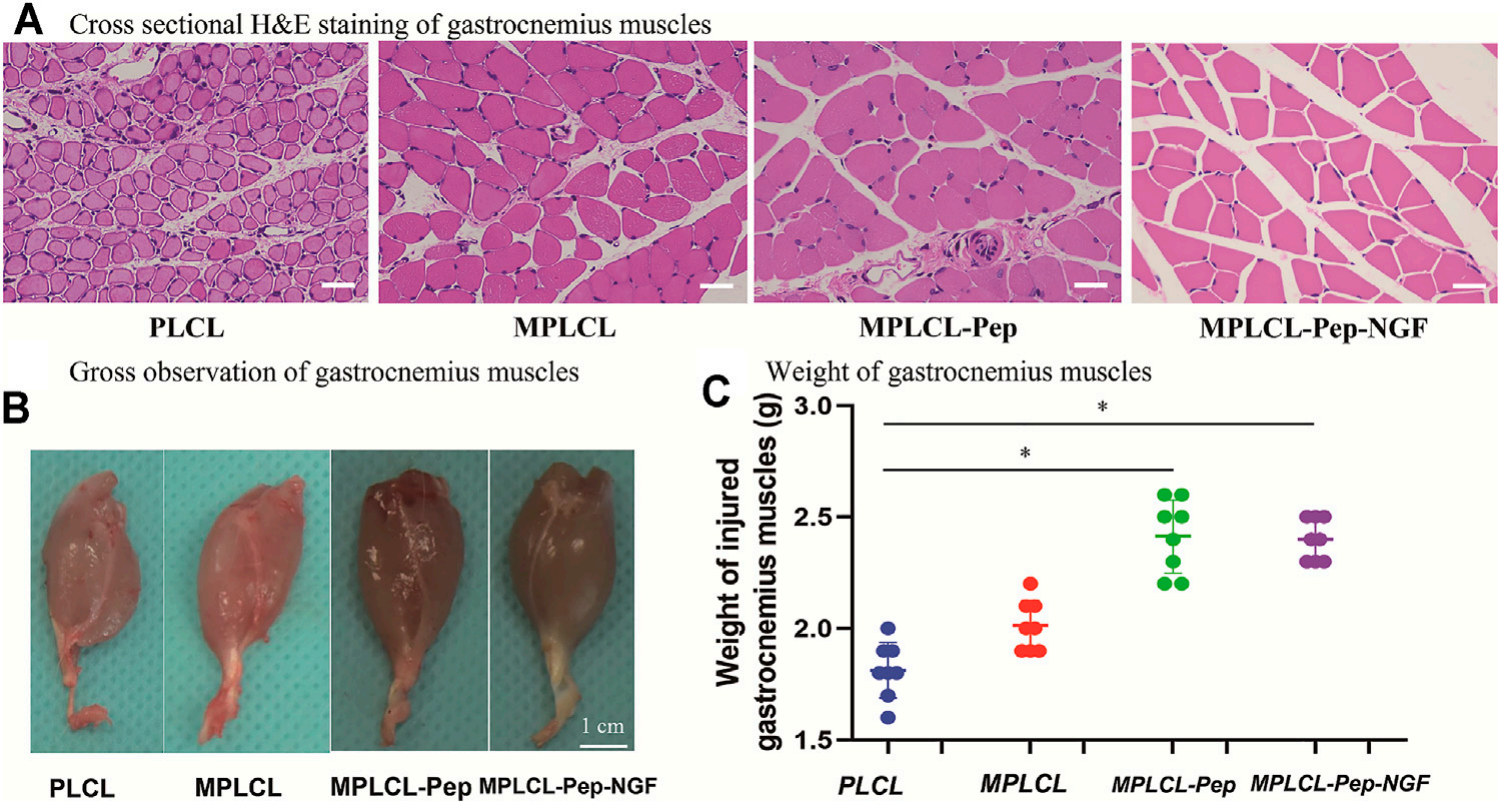
Morphological changes of gastrocnemius muscles in each treatment group of PLCL, MPLCL, MPLCL-peptides, and MPLCL-peptides-NGF. **(A)** Cross-sectional H&E staining of gastrocnemius muscles. Scale bars: 50 μm. **(B)** Gross observation of gastrocnemius muscles. Scale bars: 1 cm. **(C)** Weight of the gastrocnemius muscles. Asterisk (*) indicates statistically significant difference at *p* < 0.05 level, *n* = 8.

The macro- and micro-morphological changes of gastrocnemius muscles are recognized as a vital index to evaluate nerve functional recovery. At 4 weeks after surgery, the gross and microscopic changes of gastrocnemius muscles were observed (Figure 8). H&E staining indicates that the skeletal muscle cells have lost their cytoplasm, and the myofibers were sparse and separated by large distances with fibrosis increased and fatty infiltration in the PLCL group and MPLCL group. By contrast, the atrophic manifestation was significantly decreased in the MPLCL-peptide and MPLCL-peptide-NGF groups. The weight of gastrocnemius muscle in the MPLCL-peptide and MPLCL-peptide-NGF groups was significantly higher than that in the PLCL group, whereas there was no significant difference between the PLCL group and MPLCL group.

## Discussion

Complete recovery of neural functions after severe traction injury is a great challenge in clinic (Mahan et al., 2019). The treatment by using artificially engineered NGCs can provide an appropriate peripheral nerve growth environment *via* the improvement of physicochemical properties, surface longitudinally oriented microstructures, and bioactive molecule modification (Carvalho et al., 2019; Vijayavenkataraman, 2020). It was found that the NGCs having aligned topology and bioactive molecules on their inner surface can effectively control cell behaviors and nerve regeneration (Oh et al., 2018; Shah et al., 2019; Samadian et al., 2020). However, the effects of combination in the repair of nerve traction injury are still unknown. The aim of this work is to explore the beneficial combinations of KHI peptide modification, NGF loading, and surface linear micropatterns in the reinnervation after severe traction.

The biodegradation of PLCL is very slow and would not take place apparently within 21 days (Zhang et al., 2015; Zhang et al., 2020b). The macroprous structures on the surface of PLCL films were obtained after leaching of the NaCl porogen particles (Supplementary Figure S1). The porous structures with a suitable pore size were beneficial for cell loading and adhesion, which is consistent with previous report (Sun et al., 2021). The 3D and porous structures offer a larger surface area, enabling the efficient grafting/loading of KHI peptides and NGF. Besides, the porous structure allows better supply of nutrients and oxygen *via* the pores on the NGCs, which are extremely important for cell proliferation and nerve regeneration inside the NGCs. Nonetheless, the overall linear (grooves and ridges) pattern structure was maintained although the patterns on the porous regions were distorted or absent.

The groove and ridge morphologic surface belongs to one of the longitudinally oriented microstructures, which can affect the nerve cellular behaviors and guide the directional migration of SCs (Wang et al., 2015). It has been found that the surface anisotropic stripes of grooves and ridges can bring meaningful improvement to nerve repair by adapting the morphology, orientation, and motility of nervous system cells (Tonazzini et al., 2015). Besides, Zhang et al. (2018b) found that SCs cultured on 20/40 μm polyester migrate along the stripes, with a higher ratio of L/W compared to those on the flat film. Furthermore, the migration rate of SCs was also increased significantly when being cultured on the 3/3-μm parallel stripe PLCL, which may promote the M2 polarization of macrophage (Zhang et al., 2020a). In this study, the microstructures of parallel grooves and ridges were created on the surface of the PLCL film, which could similarly guide the direction of SCs and enhance the ability of migration even though the macroprous structure was further implemented. Moreover, these microstructures were also sculptured on the surface of NGCs and implemented in the recovery of severe nerve traction injury *in vivo*. The results reveal that the microstructures of parallel grooves and ridges on the NGCs were helpful in alleviating the nonlinear structures at the injured point after severe traction injury.

The KHI peptide (sequence Lys-His-Ile-Phe-Ser-Asp-Asp-Ser-Ser-Glu-Lys-Pra) is derived from an NCAM, which is a transmembrane protein specifically expressed on neural cells, including neuron, SCs, and other neuroglial cells (Kam et al., 2002; Turner et al., 2019). In our previous study, we developed a complementary density gradient of KHI peptides and demonstrated that the SCs spread better and directionally migrate to the direction of a higher concentration of KHI peptides (Ren et al., 2015). After KHI peptides are immobilized on substrates, the activated NCAM sites can trigger the formation of focal adhesion, improving SC migration (Maness and Schachner, 2007; Lehembre et al., 2008). Furthermore, it was also reported that the KHI peptides can promote the adhesion of neuroglial cells but not affect the adhesion of fibroblasts (Kam et al., 2002). These results prevent the consequence of aberrant reinnervation, which leads to synkinesis rather than physiologic motion (Crumley, 2000). In the present study, the KHI peptides were modified on the surface of a PLCL film, showing a beneficial effect in promoting SC adhesion *in vitro*. Moreover, the NGCs modified with KHI peptides were also firstly implemented to repair peripheral nerve traction injury *in vivo*, showing much effective in elevating the level of recovery and accelerating the process of nerve reinnervation.

The NGF is a well-known neurotrophic factor and widely used in the clinical treatment for peripheral nerve regeneration (Liu M.-Y. et al., 2020). However, a platform for controlled delivery is required because of its short half-life *in vivo* and its potential to impede axonal regeneration when used in supraphysiological doses (Lackington et al., 2019). Hence, we introduced a PLCL porous film loaded with NGF to investigate its effect on the promotion of nerve regeneration. It has been found that KHI-peptide modification did not influence on NGF release. Besides, the porous property of the films would prolong the release time of NGF till reaching 1 week. As shown in Supplementary Figure S2, the loaded NGF could be released in a sustained manner, and the cumulative dose of NGF reached up to 35 ng. The loading of NGF was found beneficial in nerve regeneration on the PLCL film having KHI peptides and micropatterns. As shown in Figure 4, the proliferation and elongation of SCs were increased significantly in the MPLCL-peptide-NGF group compared with those in the PLCL group. Furthermore, the MPLCL-peptide-NGF conduits were also found more beneficial in nerve regeneration after severe traction injury compared with the PLCL group *in vivo*. The results reveal that NGF loading is helpful to nerve reinnervation after severe traction injury, and the molecular mechanism would be investigated in our following work.

The macroprous structures, groove and ridge morphologic surface, KHI peptide, and NGF modifications were found helpful in nerve regeneration after severe traction injury *in vivo*. As shown in Figure 5, the animal model of peripheral nerve severe traction injury was validated. The injured peripheral nerves were treated by the NGCs of PLCL, MPLCL, MPLCL-peptides, and MPLCL-peptide-NGF respectively for 4 weeks. It was found that the neural EMG functions were recovered best in the group of MPLCL-peptide-NGF, including the amplitude increase and NCV acceleration (Figure 6). The gait analysis has been performed widely for evaluating the recovery of motor function as a result of functional recovery after peripheral nerve injury in the rat (Varejao et al., 2001). However, it had been reported that paw contraction and autotomy confounded the measurement of toe spread, a factor that is given particular weighting in the formula for sciatic function index determination (Ao et al., 2011). The conduction velocity is an objective and reliable index for evaluation of the conduction of action potential in peripheral nerves (Jiao et al., 2009). Therefore, we chose neural monitoring and nerve conductive velocity as the indexes to evaluate nerve functional recovery. It can be also found that the fiber spacing was reduced, and the ratio of fiber fragment was significantly decreased in the MPLCL-peptide group and MPLCL-peptide-NGF group (Figure 7). Besides, the atrophic manifestation of gastrocnemius muscle was significantly decreased in the MPLCL-peptide and MPLCL-peptide-NGF groups, showing the weight of gastrocnemius muscle gaining and the diameter of muscle bundle increasing (Figure 8). As a result, the combination of KHI peptide modification, NGF loading, and parallel linear micropattern surface could promote the reinnervation of the sciatic nerve after severe traction injury *in vivo*.

## Conclusion

The PLCL films/NGCs were successfully modified with KHI peptides, NGF loading, and parallelly linear micropatterns. The KHI peptide modification and NGF loading were beneficial in SC proliferation, migration, and adhesion. The groove and ridge morphologic surface on the NGCs were helpful in alleviating the nerve nonlinear structures at the injured point after severe traction injury. The KHI peptide modification, NGF loading, and parallel linear micropattern surface could promote the adhesion and elongation of SCs *in vitro* and the reinnervation of the sciatic nerve after severe traction injury *in vivo*. Nonetheless, the underlying molecular mechanisms need to be elucidated further in the future.

## Data Availability

The original contributions presented in the study are included in the article/Supplementary Material; further inquiries can be directed to the corresponding authors.

## Supplementary Material

Supplementary Figure S1 SEM image of NaCl particles. The average diameter was measured as 75.6 ± 19.1 μm from the SEM images.

Supplementary Figure S2 The cumulative release of NGF after 1, 3 and 7 days.
