# Supplementary figures and images for: Micropatterned Poly(D,L-Lactide-Co-Caprolactone) Conduits With KHI-Peptide and NGF Promote Peripheral Nerve Repair After Severe Traction Injury
Source: Front Bioeng Biotechnol. 2021 Dec 9;9:744230. doi: 10.3389/fbioe.2021.744230 (PMC8696012; doi:10.3389/fbioe.2021.744230)

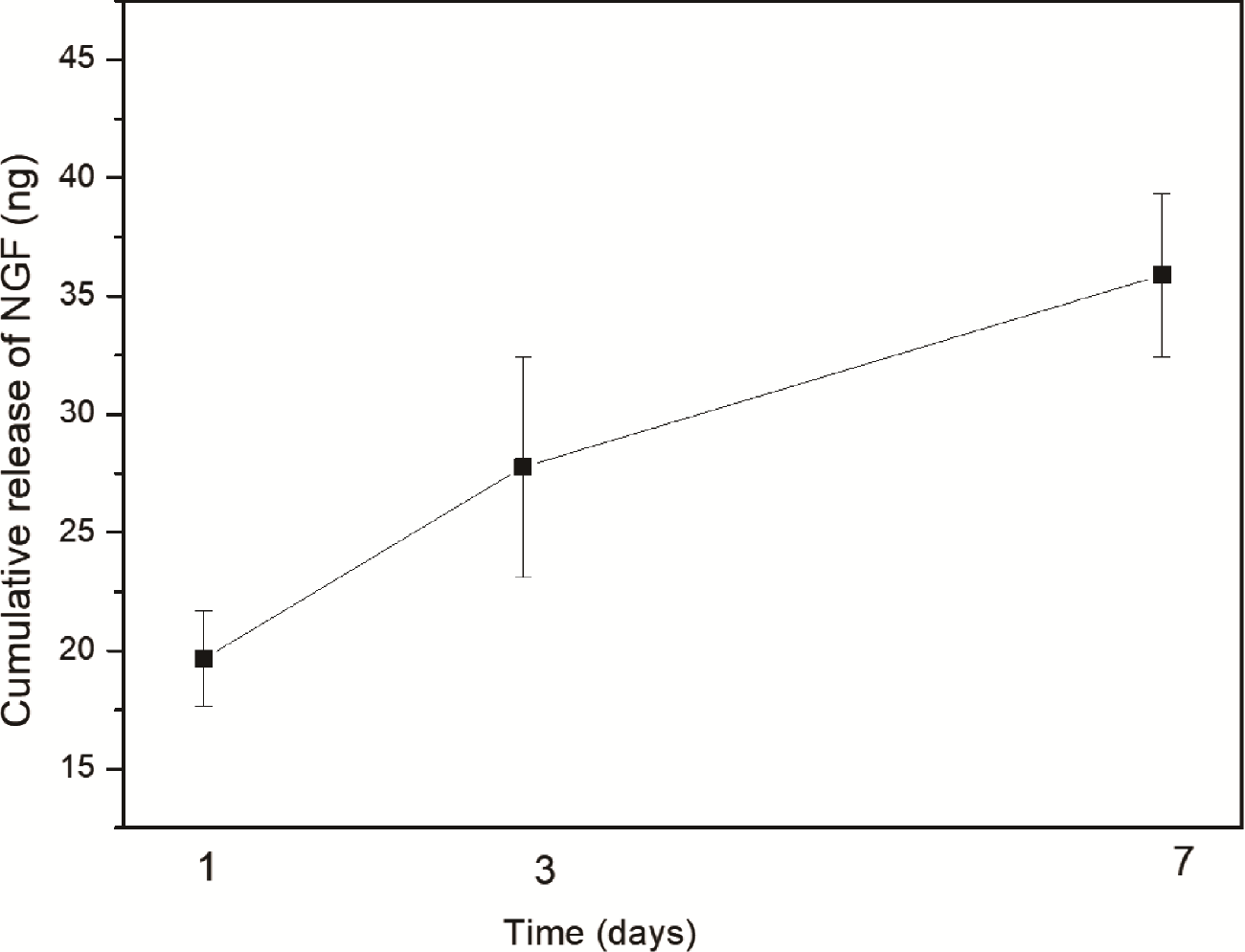

Supplement: Supplementary file 1 [file Image2.TIF]

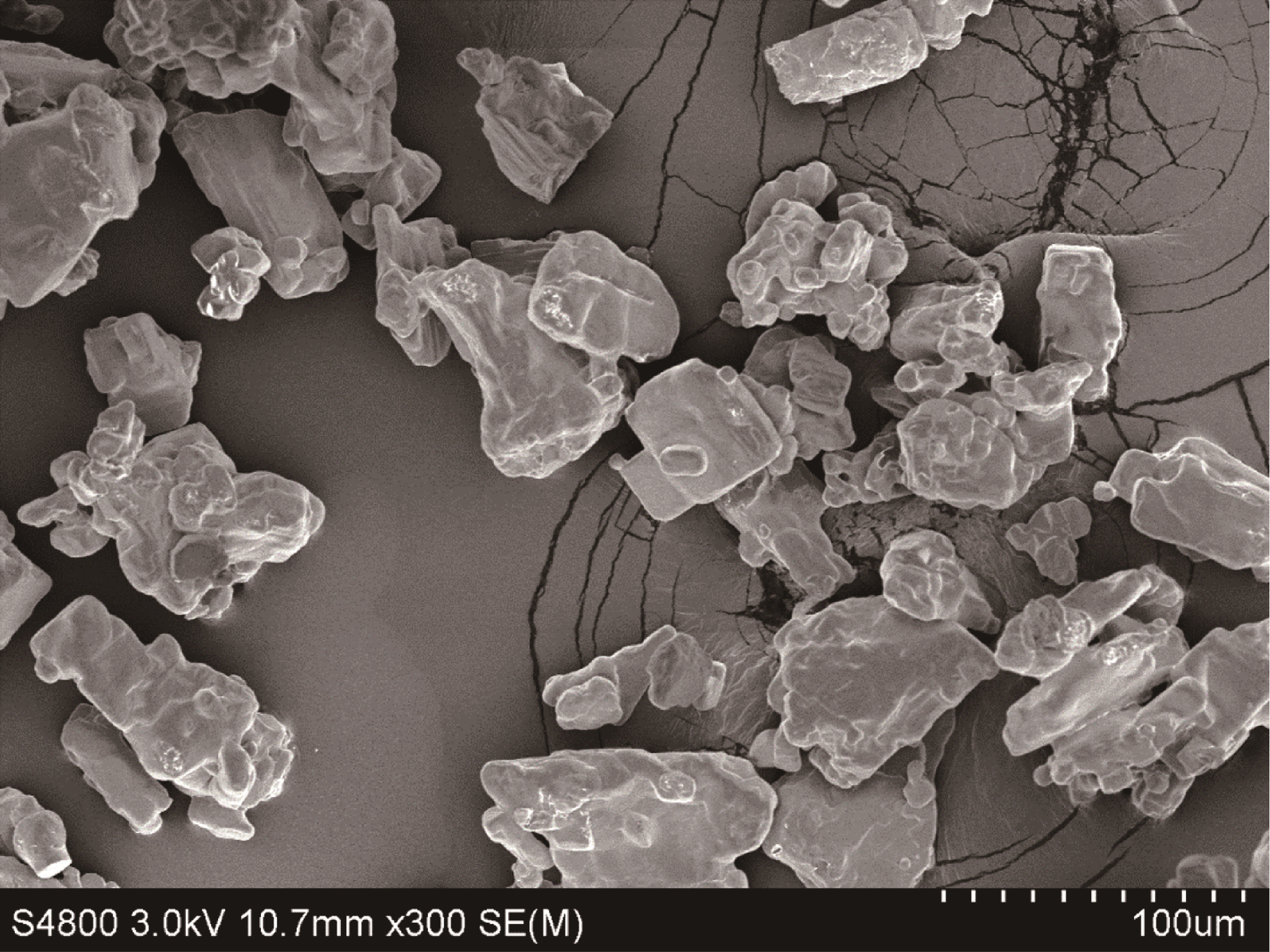

Supplement: Supplementary file 2 [file Image1.TIF]
